# (*E*)-3-[3,4-Bis(meth­oxy­methoxy)phen­yl]-1-(7-hy­droxy-5-meth­oxy-2,2-dimethyl­chroman-8-yl)prop-2-en-1-one

**DOI:** 10.1107/S160053681103131X

**Published:** 2011-08-11

**Authors:** Nur Athirah Hashim, Farediah Ahmad, Norazah Basar, Khalijah Awang, Seik Weng Ng

**Affiliations:** a Department of Chemistry, Universiti Teknologi Malaysia, 81310 Skudai, Johor, Malaysia; b Department of Chemistry, University of Malaya, 50603 Kuala Lumpur, Malaysia; c Chemistry Department, Faculty of Science, King Abdulaziz University, PO Box 80203 Jeddah, Saudi Arabia

## Abstract

The reaction of 5,6-(2,2-dimethyl­chroman­yl)-2-hy­droxy-4-meth­oxy­acetophenone and 3,4-bis­(meth­oxy­meth­yloxy)benzaldehyde affords the intense orange title chalcone derivative, C_25_H_30_O_8_. The two benzene rings are connected through a —C(=O)—CH=CH— (propenone) unit, which is in an *E* conformation; the ring with the hy­droxy substitutent is aligned at 19.5 (2)° with respect to this unit, whereas the ring with the meth­oxy­meth­yloxy substituent is aligned at 9.3 (3)°. The dihedral angle between the rings is 19.38 (10)°. The hy­droxy group engages in an intra­molecular O—H⋯O hydrogen bond with the carbonyl O atom of the propenone unit, generating an *S*(5) ring.

## Related literature

For background to chalcones, see: Avila *et al.* (2008 ▶); Narender *et al.* (2007 ▶); Reddy *et al.* (2010 ▶).
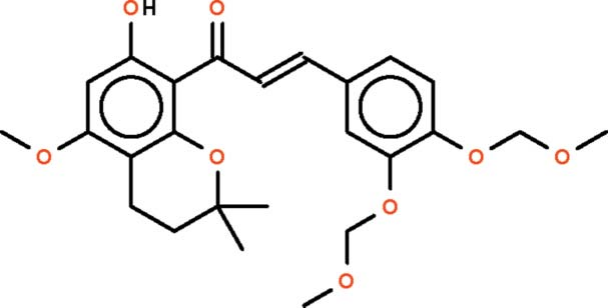


## Experimental

### 

#### Crystal data

C_25_H_30_O_8_
*M* *_r_* = 458.49
Monoclinic, 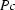

*a* = 9.5990 (8) Å
*b* = 8.3294 (7) Å
*c* = 14.7444 (12) Å
β = 107.684 (1)°
*V* = 1123.17 (16) Å^3^
*Z* = 2
Mo *K*α radiation
μ = 0.10 mm^−1^
*T* = 100 K
0.30 × 0.25 × 0.05 mm

#### Data collection

Bruker SMART APEX CCD diffractometer
10316 measured reflections
2576 independent reflections
2433 reflections with *I* > 2σ(*I*)
*R*_int_ = 0.037

#### Refinement

*R*[*F* ^2^ > 2σ(*F* ^2^)] = 0.032
*wR*(*F* ^2^) = 0.082
*S* = 1.06
2576 reflections
302 parameters
2 restraints
H atoms treated by a mixture of independent and constrained refinement
Δρ_max_ = 0.25 e Å^−3^
Δρ_min_ = −0.20 e Å^−3^

### 

Data collection: *APEX2* (Bruker, 2009 ▶); cell refinement: *SAINT* (Bruker, 2009 ▶); data reduction: *SAINT*; program(s) used to solve structure: *SHELXS97* (Sheldrick, 2008 ▶); program(s) used to refine structure: *SHELXL97* (Sheldrick, 2008 ▶); molecular graphics: *X-SEED* (Barbour, 2001 ▶); software used to prepare material for publication: *publCIF* (Westrip, 2010 ▶).

## Supplementary Material

Crystal structure: contains datablock(s) global, I. DOI: 10.1107/S160053681103131X/hb6345sup1.cif

Structure factors: contains datablock(s) I. DOI: 10.1107/S160053681103131X/hb6345Isup2.hkl

Supplementary material file. DOI: 10.1107/S160053681103131X/hb6345Isup3.cml

Additional supplementary materials:  crystallographic information; 3D view; checkCIF report

## Figures and Tables

**Table 1:** Hydrogen-bond geometry (Å, °)

*D*—H⋯*A*	*D*—H	H⋯*A*	*D*⋯*A*	*D*—H⋯*A*
O3—H3⋯O4	0.90 (4)	1.65 (4)	2.480 (2)	153 (3)

**Fig. 1.**
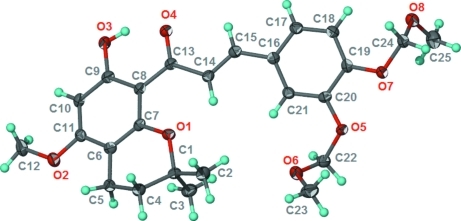
The molecular structure of (I) at the 70% probability level; hydrogen atoms are drawn as spheres of arbitrary radius.

## supplementary crystallographic information

### Comment

Chalcones or 1,3-diphenyl-2-propen-1-one derivatives flavonoids consist of two aromatic rings that are linked by a three-carbon α, β-unsaturated carbonyl unit (Avila *et al.*, 2008; Reddy *et al.*, 2010), and key precursors for the synthesis of a various flavonoids, some of which are components in food (Narender *et al.*, 2007). We intend to use the intensely-orange title compound, (I), in the synthesis of other compounds. Its two benzene rings are connected through the –C(═ O)–CH═CH– unit, which is of an *E* configuration; the ring with the hydroxy substitutent is aligned at 19.5 (2) ° with this unit whereas the ring with the methoxymethyloxy substituents is aligned at 9.3 (3) °. The hydroxy group engages in intramolecular hydrogen bonding with the carbonyl O atom of the unit (Fig.1).

### Experimental

A solution of 2-hydroxy-4-methoxy-5,6-(2,2-dimethylchromane)acetophenone (100 mg, 0.45 mmol) and 3,4-bis(methoxymethyloxy)benzaldehyde (100 mg, 0.45 mmol) in ethanol (10 ml) was treated with 50% potassium hydroxide (1 ml). The mixture was stirred for 48 h. The mixture was poured into iced water (30 ml); this was acidified with 10% hydrochloric acid. The mixture was extracted with dichloromethane (3 *x* 20 ml). The organic layer was washed with water (3 *x* 10 ml) and brine (3 *x* 5 ml) followed by drying over anhydrous magnesium sulfate. The solvent was evaporated under reduced pressure to yield a dark greenish syrup. The syrup was subjected to VLC for purification by using silica gel and eluting with a hexane:ethyl acetate solvent system (9:1) to give the title compound (520 mg, 30%) as orange prisms of (I), m.p. 363–368 K. The formulation was established by ^1^H– and ^13^C-NMR spectroscopy.

### Refinement

Carbon-bound H-atoms were placed in calculated positions (C—H 0.95 to 0.98 Å) and were included in the refinement in the riding model approximation, with *U*(H) set to 1.2 to 1.5*U*_eq_(C).

The hydroxy H-atom was located in a difference Fourier map, and was freely refined.

In the absence of heavy scatters, 2245 Friedel pairs were merged. Omitted from the refinement were (-3 3 - 8), (-2 8 - 1), (1 1 - 4), (-4 9 3) and (-3 0 16).

### Crystal data

**Table:**

C_25_H_30_O_8_	*F*(000) = 488
*M**_r_* = 458.49	*D*_x_ = 1.356 Mg m^−^^3^
Monoclinic, *P**c*	Mo *K*α radiation, λ = 0.71073 Å
Hall symbol: P -2yc	Cell parameters from 3857 reflections
*a* = 9.5990 (8) Å	θ = 2.2–28.2°
*b* = 8.3294 (7) Å	µ = 0.10 mm^−^^1^
*c* = 14.7444 (12) Å	*T* = 100 K
β = 107.684 (1)°	Prism, orange
*V* = 1123.17 (16) Å^3^	0.30 × 0.25 × 0.05 mm
*Z* = 2	

### Data collection

**Table:**

Bruker SMART APEX CCD diffractometer	2433 reflections with *I* > 2σ(*I*)
Radiation source: fine-focus sealed tube	*R*_int_ = 0.037
graphite	θ_max_ = 27.5°, θ_min_ = 2.2°
ω scans	*h* = −12→12
10316 measured reflections	*k* = −10→10
2576 independent reflections	*l* = −19→19

### Refinement

**Table:**

Refinement on *F*^2^	Primary atom site location: structure-invariant direct methods
Least-squares matrix: full	Secondary atom site location: difference Fourier map
*R*[*F*^2^ > 2σ(*F*^2^)] = 0.032	Hydrogen site location: inferred from neighbouring sites
*wR*(*F*^2^) = 0.082	H atoms treated by a mixture of independent and constrained refinement
*S* = 1.06	*w* = 1/[σ^2^(*F*_o_^2^) + (0.0491*P*)^2^ + 0.1273*P*] where *P* = (*F*_o_^2^ + 2*F*_c_^2^)/3
2576 reflections	(Δ/σ)_max_ = 0.001
302 parameters	Δρ_max_ = 0.25 e Å^−^^3^
2 restraints	Δρ_min_ = −0.20 e Å^−^^3^

### Fractional atomic coordinates and isotropic or equivalent isotropic displacement parameters (Å^2^)

**Table:**

	*x*	*y*	*z*	*U*_iso_*/*U*_eq_	
O1	0.50254 (17)	0.07085 (17)	0.49935 (10)	0.0174 (3)	
O2	0.69328 (17)	0.04190 (19)	0.83437 (11)	0.0204 (3)	
O3	0.79441 (18)	0.4985 (2)	0.67354 (12)	0.0221 (3)	
H3	0.777 (4)	0.526 (4)	0.612 (3)	0.042 (9)*	
O4	0.68542 (17)	0.51474 (19)	0.49826 (11)	0.0198 (3)	
O5	0.01705 (16)	0.23344 (18)	0.10555 (10)	0.0178 (3)	
O6	0.06369 (19)	0.0284 (2)	0.22016 (12)	0.0251 (4)	
O7	0.05288 (16)	0.41671 (18)	−0.02881 (10)	0.0187 (3)	
O8	0.05354 (17)	0.66810 (19)	−0.10124 (11)	0.0210 (3)	
C1	0.4074 (2)	−0.0676 (2)	0.49859 (16)	0.0181 (4)	
C2	0.3732 (3)	−0.1334 (3)	0.39786 (16)	0.0237 (5)	
H2A	0.4635	−0.1720	0.3874	0.036*	
H2B	0.3304	−0.0482	0.3520	0.036*	
H2C	0.3036	−0.2224	0.3894	0.036*	
C3	0.2692 (2)	−0.0069 (3)	0.51717 (18)	0.0228 (5)	
H3A	0.2199	0.0701	0.4675	0.034*	
H3B	0.2949	0.0460	0.5795	0.034*	
H3C	0.2038	−0.0975	0.5165	0.034*	
C4	0.4918 (2)	−0.1879 (3)	0.57238 (15)	0.0194 (4)	
H4A	0.5793	−0.2241	0.5559	0.023*	
H4B	0.4295	−0.2829	0.5718	0.023*	
C5	0.5390 (2)	−0.1138 (3)	0.67197 (15)	0.0193 (4)	
H5A	0.4542	−0.1096	0.6969	0.023*	
H5B	0.6152	−0.1819	0.7153	0.023*	
C6	0.5981 (2)	0.0529 (2)	0.66954 (15)	0.0160 (4)	
C7	0.5763 (2)	0.1365 (3)	0.58478 (15)	0.0149 (4)	
C8	0.6366 (2)	0.2927 (3)	0.58144 (15)	0.0148 (4)	
C9	0.7257 (2)	0.3568 (3)	0.66940 (15)	0.0166 (4)	
C10	0.7437 (2)	0.2780 (3)	0.75539 (15)	0.0174 (4)	
H10	0.7988	0.3259	0.8137	0.021*	
C11	0.6798 (2)	0.1281 (3)	0.75453 (15)	0.0161 (4)	
C12	0.7820 (3)	0.1083 (3)	0.92334 (15)	0.0225 (5)	
H12A	0.7826	0.0346	0.9753	0.034*	
H12B	0.7416	0.2120	0.9342	0.034*	
H12C	0.8822	0.1233	0.9211	0.034*	
C13	0.6066 (2)	0.3942 (3)	0.49629 (14)	0.0150 (4)	
C14	0.4826 (2)	0.3661 (3)	0.41004 (15)	0.0163 (4)	
H14	0.4162	0.2807	0.4081	0.020*	
C15	0.4642 (2)	0.4620 (3)	0.33424 (15)	0.0167 (4)	
H15	0.5348	0.5446	0.3407	0.020*	
C16	0.3495 (2)	0.4553 (3)	0.24339 (15)	0.0160 (4)	
C17	0.3573 (2)	0.5633 (2)	0.17258 (15)	0.0178 (4)	
H17	0.4318	0.6428	0.1869	0.021*	
C18	0.2588 (2)	0.5565 (3)	0.08200 (15)	0.0174 (4)	
H18	0.2660	0.6310	0.0349	0.021*	
C19	0.1493 (2)	0.4408 (3)	0.05985 (14)	0.0163 (4)	
C20	0.1330 (2)	0.3372 (2)	0.13222 (15)	0.0147 (4)	
C21	0.2341 (2)	0.3429 (2)	0.22193 (15)	0.0159 (4)	
H21	0.2257	0.2703	0.2697	0.019*	
C22	−0.0247 (2)	0.1575 (3)	0.18001 (15)	0.0201 (4)	
H22A	−0.0210	0.2375	0.2303	0.024*	
H22B	−0.1270	0.1199	0.1543	0.024*	
C23	0.0360 (3)	−0.1112 (3)	0.16131 (19)	0.0282 (5)	
H23A	0.1017	−0.1979	0.1934	0.042*	
H23B	0.0531	−0.0866	0.1006	0.042*	
H23C	−0.0657	−0.1451	0.1496	0.042*	
C24	0.0800 (2)	0.5048 (3)	−0.10506 (15)	0.0178 (4)	
H24A	0.1831	0.4887	−0.1030	0.021*	
H24B	0.0171	0.4616	−0.1664	0.021*	
C25	−0.0952 (3)	0.7036 (3)	−0.10670 (18)	0.0271 (5)	
H25A	−0.1074	0.8200	−0.1034	0.041*	
H25B	−0.1188	0.6520	−0.0535	0.041*	
H25C	−0.1607	0.6630	−0.1670	0.041*	

### Atomic displacement parameters (Å^2^)

**Table:**

	*U*^11^	*U*^22^	*U*^33^	*U*^12^	*U*^13^	*U*^23^
O1	0.0221 (8)	0.0131 (7)	0.0157 (7)	−0.0047 (6)	0.0039 (6)	−0.0013 (6)
O2	0.0239 (8)	0.0202 (8)	0.0156 (7)	−0.0001 (6)	0.0038 (6)	0.0013 (6)
O3	0.0244 (8)	0.0185 (8)	0.0178 (8)	−0.0070 (6)	−0.0018 (6)	0.0009 (6)
O4	0.0205 (8)	0.0183 (8)	0.0175 (7)	−0.0055 (6)	0.0013 (6)	0.0006 (6)
O5	0.0174 (7)	0.0182 (8)	0.0161 (7)	−0.0057 (6)	0.0026 (6)	0.0007 (6)
O6	0.0279 (9)	0.0205 (8)	0.0221 (8)	−0.0044 (7)	0.0004 (7)	0.0051 (6)
O7	0.0202 (8)	0.0189 (8)	0.0149 (7)	−0.0030 (6)	0.0023 (6)	0.0022 (6)
O8	0.0227 (8)	0.0182 (8)	0.0206 (8)	0.0002 (6)	0.0044 (7)	0.0026 (6)
C1	0.0199 (11)	0.0120 (10)	0.0202 (10)	−0.0048 (8)	0.0030 (8)	−0.0014 (8)
C2	0.0278 (12)	0.0180 (11)	0.0216 (11)	−0.0053 (9)	0.0019 (10)	−0.0033 (9)
C3	0.0165 (10)	0.0197 (10)	0.0313 (12)	0.0002 (8)	0.0061 (9)	0.0020 (9)
C4	0.0200 (11)	0.0128 (10)	0.0226 (11)	−0.0019 (8)	0.0024 (9)	0.0000 (8)
C5	0.0209 (11)	0.0150 (10)	0.0191 (11)	−0.0016 (8)	0.0020 (9)	0.0025 (8)
C6	0.0150 (10)	0.0137 (10)	0.0192 (10)	0.0007 (8)	0.0050 (8)	0.0002 (8)
C7	0.0116 (9)	0.0144 (10)	0.0179 (10)	0.0010 (7)	0.0032 (8)	−0.0017 (8)
C8	0.0133 (10)	0.0140 (10)	0.0165 (9)	0.0000 (8)	0.0035 (8)	−0.0017 (7)
C9	0.0146 (10)	0.0151 (10)	0.0194 (11)	0.0007 (8)	0.0039 (8)	−0.0004 (8)
C10	0.0149 (10)	0.0189 (10)	0.0161 (10)	0.0004 (8)	0.0010 (8)	−0.0016 (8)
C11	0.0134 (10)	0.0181 (10)	0.0167 (10)	0.0031 (8)	0.0044 (8)	0.0030 (8)
C12	0.0289 (12)	0.0225 (11)	0.0134 (10)	0.0044 (10)	0.0022 (9)	0.0010 (9)
C13	0.0143 (10)	0.0139 (10)	0.0167 (10)	0.0004 (8)	0.0046 (8)	−0.0009 (7)
C14	0.0162 (10)	0.0134 (10)	0.0179 (10)	−0.0016 (8)	0.0030 (8)	−0.0014 (8)
C15	0.0178 (10)	0.0138 (10)	0.0188 (10)	−0.0012 (8)	0.0058 (8)	−0.0023 (8)
C16	0.0167 (10)	0.0150 (10)	0.0169 (10)	−0.0001 (8)	0.0058 (8)	−0.0005 (8)
C17	0.0203 (11)	0.0139 (10)	0.0189 (10)	−0.0024 (8)	0.0055 (8)	−0.0005 (8)
C18	0.0190 (10)	0.0162 (11)	0.0172 (10)	−0.0007 (8)	0.0059 (8)	0.0018 (8)
C19	0.0189 (10)	0.0152 (10)	0.0140 (10)	0.0018 (8)	0.0037 (8)	−0.0006 (8)
C20	0.0156 (10)	0.0106 (9)	0.0189 (10)	−0.0005 (8)	0.0065 (8)	−0.0020 (8)
C21	0.0166 (10)	0.0143 (10)	0.0170 (10)	0.0006 (8)	0.0054 (8)	0.0016 (8)
C22	0.0206 (11)	0.0229 (11)	0.0169 (10)	−0.0047 (9)	0.0057 (9)	−0.0004 (8)
C23	0.0282 (13)	0.0222 (12)	0.0345 (14)	−0.0057 (10)	0.0099 (11)	−0.0002 (10)
C24	0.0186 (10)	0.0193 (11)	0.0149 (10)	0.0002 (8)	0.0043 (8)	0.0020 (8)
C25	0.0267 (13)	0.0280 (12)	0.0260 (12)	0.0072 (10)	0.0070 (10)	0.0006 (10)

### Geometric parameters (Å, °)

**Table:**

O1—C7	1.358 (3)	C8—C9	1.423 (3)
O1—C1	1.469 (2)	C8—C13	1.467 (3)
O2—C11	1.351 (3)	C9—C10	1.391 (3)
O2—C12	1.440 (3)	C10—C11	1.390 (3)
O3—C9	1.344 (3)	C10—H10	0.9500
O3—H3	0.90 (4)	C12—H12A	0.9800
O4—C13	1.253 (3)	C12—H12B	0.9800
O5—C20	1.369 (2)	C12—H12C	0.9800
O5—C22	1.426 (2)	C13—C14	1.472 (3)
O6—C22	1.387 (3)	C14—C15	1.341 (3)
O6—C23	1.427 (3)	C14—H14	0.9500
O7—C19	1.368 (3)	C15—C16	1.454 (3)
O7—C24	1.431 (2)	C15—H15	0.9500
O8—C24	1.388 (3)	C16—C17	1.397 (3)
O8—C25	1.436 (3)	C16—C21	1.410 (3)
C1—C4	1.519 (3)	C17—C18	1.384 (3)
C1—C3	1.520 (3)	C17—H17	0.9500
C1—C2	1.523 (3)	C18—C19	1.389 (3)
C2—H2A	0.9800	C18—H18	0.9500
C2—H2B	0.9800	C19—C20	1.418 (3)
C2—H2C	0.9800	C20—C21	1.383 (3)
C3—H3A	0.9800	C21—H21	0.9500
C3—H3B	0.9800	C22—H22A	0.9900
C3—H3C	0.9800	C22—H22B	0.9900
C4—C5	1.529 (3)	C23—H23A	0.9800
C4—H4A	0.9900	C23—H23B	0.9800
C4—H4B	0.9900	C23—H23C	0.9800
C5—C6	1.504 (3)	C24—H24A	0.9900
C5—H5A	0.9900	C24—H24B	0.9900
C5—H5B	0.9900	C25—H25A	0.9800
C6—C7	1.390 (3)	C25—H25B	0.9800
C6—C11	1.407 (3)	C25—H25C	0.9800
C7—C8	1.431 (3)		
			
C7—O1—C1	118.07 (16)	O2—C12—H12B	109.5
C11—O2—C12	117.60 (17)	H12A—C12—H12B	109.5
C9—O3—H3	104 (2)	O2—C12—H12C	109.5
C20—O5—C22	116.93 (16)	H12A—C12—H12C	109.5
C22—O6—C23	113.33 (18)	H12B—C12—H12C	109.5
C19—O7—C24	116.34 (16)	O4—C13—C8	118.78 (18)
C24—O8—C25	113.00 (18)	O4—C13—C14	118.10 (18)
O1—C1—C4	108.51 (17)	C8—C13—C14	123.03 (18)
O1—C1—C3	108.19 (17)	C15—C14—C13	119.54 (19)
C4—C1—C3	113.25 (18)	C15—C14—H14	120.2
O1—C1—C2	103.86 (16)	C13—C14—H14	120.2
C4—C1—C2	111.78 (18)	C14—C15—C16	128.0 (2)
C3—C1—C2	110.74 (19)	C14—C15—H15	116.0
C1—C2—H2A	109.5	C16—C15—H15	116.0
C1—C2—H2B	109.5	C17—C16—C21	118.45 (19)
H2A—C2—H2B	109.5	C17—C16—C15	117.75 (19)
C1—C2—H2C	109.5	C21—C16—C15	123.77 (18)
H2A—C2—H2C	109.5	C18—C17—C16	121.24 (19)
H2B—C2—H2C	109.5	C18—C17—H17	119.4
C1—C3—H3A	109.5	C16—C17—H17	119.4
C1—C3—H3B	109.5	C17—C18—C19	120.12 (19)
H3A—C3—H3B	109.5	C17—C18—H18	119.9
C1—C3—H3C	109.5	C19—C18—H18	119.9
H3A—C3—H3C	109.5	O7—C19—C18	124.78 (18)
H3B—C3—H3C	109.5	O7—C19—C20	115.63 (18)
C1—C4—C5	111.09 (18)	C18—C19—C20	119.59 (19)
C1—C4—H4A	109.4	O5—C20—C21	124.72 (18)
C5—C4—H4A	109.4	O5—C20—C19	115.73 (18)
C1—C4—H4B	109.4	C21—C20—C19	119.53 (18)
C5—C4—H4B	109.4	C20—C21—C16	120.83 (18)
H4A—C4—H4B	108.0	C20—C21—H21	119.6
C6—C5—C4	110.70 (17)	C16—C21—H21	119.6
C6—C5—H5A	109.5	O6—C22—O5	113.22 (18)
C4—C5—H5A	109.5	O6—C22—H22A	108.9
C6—C5—H5B	109.5	O5—C22—H22A	108.9
C4—C5—H5B	109.5	O6—C22—H22B	108.9
H5A—C5—H5B	108.1	O5—C22—H22B	108.9
C7—C6—C11	117.93 (19)	H22A—C22—H22B	107.7
C7—C6—C5	121.93 (19)	O6—C23—H23A	109.5
C11—C6—C5	120.13 (18)	O6—C23—H23B	109.5
O1—C7—C6	121.74 (18)	H23A—C23—H23B	109.5
O1—C7—C8	115.87 (17)	O6—C23—H23C	109.5
C6—C7—C8	122.31 (19)	H23A—C23—H23C	109.5
C9—C8—C7	116.57 (18)	H23B—C23—H23C	109.5
C9—C8—C13	118.11 (18)	O8—C24—O7	113.05 (17)
C7—C8—C13	125.21 (18)	O8—C24—H24A	109.0
O3—C9—C10	116.71 (19)	O7—C24—H24A	109.0
O3—C9—C8	121.45 (19)	O8—C24—H24B	109.0
C10—C9—C8	121.84 (19)	O7—C24—H24B	109.0
C9—C10—C11	118.90 (19)	H24A—C24—H24B	107.8
C9—C10—H10	120.5	O8—C25—H25A	109.5
C11—C10—H10	120.5	O8—C25—H25B	109.5
O2—C11—C10	122.98 (19)	H25A—C25—H25B	109.5
O2—C11—C6	114.77 (18)	O8—C25—H25C	109.5
C10—C11—C6	122.24 (19)	H25A—C25—H25C	109.5
O2—C12—H12A	109.5	H25B—C25—H25C	109.5
			
C7—O1—C1—C4	−47.8 (2)	C5—C6—C11—C10	176.19 (19)
C7—O1—C1—C3	75.5 (2)	C9—C8—C13—O4	16.7 (3)
C7—O1—C1—C2	−166.83 (18)	C7—C8—C13—O4	−167.3 (2)
O1—C1—C4—C5	60.1 (2)	C9—C8—C13—C14	−159.84 (19)
C3—C1—C4—C5	−60.0 (2)	C7—C8—C13—C14	16.2 (3)
C2—C1—C4—C5	174.09 (18)	O4—C13—C14—C15	4.9 (3)
C1—C4—C5—C6	−43.8 (2)	C8—C13—C14—C15	−178.5 (2)
C4—C5—C6—C7	14.6 (3)	C13—C14—C15—C16	−179.6 (2)
C4—C5—C6—C11	−164.52 (19)	C14—C15—C16—C17	−176.5 (2)
C1—O1—C7—C6	18.7 (3)	C14—C15—C16—C21	1.3 (3)
C1—O1—C7—C8	−164.55 (17)	C21—C16—C17—C18	−2.9 (3)
C11—C6—C7—O1	177.99 (18)	C15—C16—C17—C18	175.06 (19)
C5—C6—C7—O1	−1.2 (3)	C16—C17—C18—C19	0.0 (3)
C11—C6—C7—C8	1.5 (3)	C24—O7—C19—C18	7.9 (3)
C5—C6—C7—C8	−177.66 (19)	C24—O7—C19—C20	−171.75 (17)
O1—C7—C8—C9	−174.29 (17)	C17—C18—C19—O7	−175.3 (2)
C6—C7—C8—C9	2.4 (3)	C17—C18—C19—C20	4.3 (3)
O1—C7—C8—C13	9.6 (3)	C22—O5—C20—C21	17.4 (3)
C6—C7—C8—C13	−173.71 (19)	C22—O5—C20—C19	−164.53 (18)
C7—C8—C9—O3	176.20 (19)	O7—C19—C20—O5	−4.1 (3)
C13—C8—C9—O3	−7.4 (3)	C18—C19—C20—O5	176.19 (18)
C7—C8—C9—C10	−5.1 (3)	O7—C19—C20—C21	174.01 (18)
C13—C8—C9—C10	171.26 (19)	C18—C19—C20—C21	−5.7 (3)
O3—C9—C10—C11	−177.43 (19)	O5—C20—C21—C16	−179.29 (19)
C8—C9—C10—C11	3.8 (3)	C19—C20—C21—C16	2.7 (3)
C12—O2—C11—C10	−1.9 (3)	C17—C16—C21—C20	1.5 (3)
C12—O2—C11—C6	176.82 (18)	C15—C16—C21—C20	−176.33 (19)
C9—C10—C11—O2	179.01 (19)	C23—O6—C22—O5	−75.0 (2)
C9—C10—C11—C6	0.4 (3)	C20—O5—C22—O6	−79.4 (2)
C7—C6—C11—O2	178.28 (17)	C25—O8—C24—O7	−60.4 (2)
C5—C6—C11—O2	−2.6 (3)	C19—O7—C24—O8	−70.3 (2)
C7—C6—C11—C10	−3.0 (3)		

### Hydrogen-bond geometry (Å, °)

**Table:**

*D*—H···*A*	*D*—H	H···*A*	*D*···*A*	*D*—H···*A*
O3—H3···O4	0.90 (4)	1.65 (4)	2.480 (2)	153 (3)
